# Informed consent in Sri Lanka: A survey among ethics committee members

**DOI:** 10.1186/1472-6939-9-10

**Published:** 2008-05-20

**Authors:** Athula Sumathipala, Sisira Siribaddana, Suwin Hewage, Manura Lekamwattage, Manjula Athukorale, Chesmal Siriwardhana, Joanna Murray, Martin Prince

**Affiliations:** 1 Kings College, University of London, Institute of Psychiatry, De Crespigny Park, Denmark Hill, London SE5 8AF, UK; 2 Institute of Research and Development, 762/4B Pannipitiya Rd, Battaramulla 10120, Sri Lanka

## Abstract

**Background:**

Approval of the research proposal by an ethical review committee from both sponsoring and host countries is a generally agreed requirement in externally sponsored research.

However, capacity for ethics review is not universal. Aim of this study was to identify opinions and views of the members serving in ethical review and ethics committees in Sri Lanka on informed consent, essential components in the information leaflet and the consent form.

**Methods:**

We obtained ethical approval from UK and Sri Lanka. A series of consensus generation meetings on the protocol were conducted. A task oriented interview guide was developed. The interview was based on open-ended questionnaire. Then the participants were given a WHO checklist on informed consent and requested to rate the items on a three point scale ranging from extremely important to not important.

**Results:**

Twenty-nine members from ethics committees participated. Majority of participants (23), believed a copy of the information leaflet and consent form, should accompany research proposal. Opinions about the items that should be included in the information leaflets varied. Participants identified 18 criteria as requirements in the information leaflet and 19 for the consent form.

The majority, 20 (69%), believed that all research need ethical approval but identified limited human resource, time and inadequate capacity as constraints. Fifteen (52%) believed that written consent is not required for all research. Verbal consent emerged as an alternative to written consent. The majority of participants rated all components of the WHO checklist as important.

**Conclusion:**

The number of themes generated for the consent form (N = 18) is as many as for the information leaflet (N = 19) and had several overlaps. This suggests that the consent form should be itemized to reflect the contents covered in the information leaflet. The participants' opinion on components of the information leaflets and consent forms proved to be similar with WHO checklist on informed consent.

## Background

Ethical Review Committees (ERC) reviews research and Ethics Committees (EC) carry out other ethics related activities. There were ERCs in 5 out of 7 medical schools in Sri Lanka at the time of conducting this research [1]. In addition, there were three ERCs and two ECs in professional associations and research institutions. Medical faculties at Colombo and Peradeniya universities were the first institutions to establish formal ERCs in late 1970's [1]. ERCs' primary duty is participant protection while sustaining research integrity [2,3]. Review and approval by an ERC is mandatory for research [4]. Reputable academic institutions in the developed world now insist ethical approval from both sponsoring and host countries for collaborative research [5,6]. However, ethics review capacity is not universally the same. Even if it is, demanding universal standards have met with opposing arguments especially in the standard of care issue [5].

Consent is considered 'informed' when given by a person who understands the purpose and the nature of research, what is required from the participant, what may be the potential benefits, and the risks resulting from the study [7,8]. Nevertheless, the question is; have they 'understood' enough about the research before giving consent? A number of studies in industrialised countries have shown the existence of gaps in the information provided and the understanding of research participants [9]. It would be fair to assume that this issue is more pronounced in a developing country setting where poverty, low literacy and large needs gap make the participants more vulnerable to exploitation [10].

We have carried out international collaborative research with UK since 1997 [11]. One recent project was collaboration between Sri Lankan Twin Registry (SLTR), Institute of Psychiatry (IoP) King's College, London and WHO. We applied for ethical approval from ERCs of University of Sri Jayewardenepura-Sri Lanka, Institute of Psychiatry, London and WHO. The details expected in the information leaflet and the consent forms were different in all three institutions.

These differences prompted us to examine the issue of consent by obtaining the ERC members' views about the subject. There is an increased need to carry out research in developing south to reduce 10/90 gap in research. Although ethical principles are universal, empirical research into ethics and its practices are important to understand local context and sensitivities. This is especially true with increased globalisation of research and health. Limited research has been carried out examining procedures, strengths and challenges facing ERCs in developing countries [12].

This study specifically aimed to examine current practices and views of ERC and EC members on the issue of informed consent in the local context and in comparison to international requirement.

This research was conducted as part of a larger study on informed consent in Sri Lanka [13]. The objective was to obtain the opinions and views of the individual ERC members on 'essential components of an information leaflet and a consent form', based on their experience.

## Methodology

### Protocol development process

Along with the other components of the project, the initial protocol of this research was subjected to revision based on the comments received from the reviewers of the funding body. There was also a consultation meeting held in UK to finalise the protocol. The proposal was also presented to an invited audience in Sri Lanka who were either involved in ethics review process or who had a special interest in ethics. Some of the participants included those who were trained during the intensive course in bioethics funded by the Wellcome Trust in 2003 [14].

Following a two-day workshop on qualitative research methods, focus group meetings on the protocol were held to develop consensus. Detailed protocol along with questionnaire was subjected to refinement in these meetings. We developed a task oriented interview guide to be used by the interviewers [see Additional file 1].

### Strategy for data collection

There are about 60 members in 10 ERCs and ECs. It was agreed during the consensus meetings that;

1. It would be adequate to recruit approximately 20 members from existing ERCs and ECs, as this is a qualitative study.

2. To over sample lay members.

3. To conduct multi-level sampling from different backgrounds.

4. To send a covering letter along with an information leaflet to all the members of ERCs and ECs.

5. To recruit participants by telephone in order to reach the appropriate number.

6. To establish rapport with the participants and clarify the research proposal as required.

### Ethical aspects

Ethical approval for the project was obtained from ERC of the Faculty of Medicine, Sri Jayewardenepura University, Sri Lanka and the Institute of Psychiatry, King's College, University of London.

Number of individuals in ERCs and ECs in Sri Lanka is small and individually known in the academic circles. Hence, the data when presented are not directly linked to the participants or their individual committees to preserve anonymity.

While we acknowledge the important intellectual contributions made by the participants, we did not name them in the acknowledgement section, as we did not obtain permission to do so when initial consent was obtained.

### Research process

A purposive sampling method was used in the selection of respondents from ERCs. More than 30 individuals were approached initially as some may not be available or agree to participate. The only selection criteria was that participants had to be currently serving members or who had served in the recent past in either ERCs or ECs, agree and available to be interviewed.

Interviewers initially setup an appointment with each member requesting their participation in the study. The research project and what was expected from them were explained. Then we provided an information leaflet about the project and the opportunity to contact the main investigators if further clarifications were required.

Some directly consented and participated in the interview. Some requested authorization for participation from the ERC they represented. This resulted in formal approval being requested from some of the ERCs.

Some participants, although they had consented to take part, had to be excluded from the study, as they could not be contacted subsequently due to unavailability.

The interview began with open-ended questions inquiring participants' opinion about components in the information leaflet and the consent form. The next part of the questionnaire contained components of WHO Secretariat Committee on Research Involving Human Subjects (SCRIHS) checklist on information leaflets (see Additional file 1, Appendix 2). These components were rated on a 4 point Likert scale (ranging from extremely important to not important) about their relevance to the local context. Multiple responses were permitted in answering open-ended questions. Two research assistants conducted the interviews and collected the data, using the interview guide. These interviews were conducted over a period covering the latter part of 2005 and early 2006.

The answers to multiple-choice questions were entered and analysed using quantitative methods, while the answers to open ended questions were entered in SPSS using 'string' value and then coded manually after pasting on a word file.

## Results

### Participants

Twenty-nine participated in the study, exceeding the sample size of 20 decided as sufficient during the consensus meetings. They were drawn from approximately 60 members of five ERCs and one EC that functioned in Sri Lanka at the time of the study. Medical as well as non-medical members were interviewed. The strategy of over sampling of lay members did not succeed as most ERCs had none or very few lay members.

Members of the ERCs from Sri Jayewardenepura (5), Ruhuna (3), Peradeniya (5), Kelaniya (5) and Colombo (1) medical faculties and the Medical Research Institute (5) and members of the EC of Sri Lanka Association for the Advancement of Science -SLAAS- (2) and Sri Lanka Medical Association (3) were interviewed. Members in two ERCs neither declined nor participated.

Participants in this study were professionals from diverse fields such as Parasitology, Sociology, Zoology, Biochemistry, Psychiatry, Community Medicine, Physiology, Microbiology, Pathology, Biotechnology, Veterinary Medicine, Forensic Medicine and Pharmacology. Five participants were microbiologists, making it the most represented area while Community Medicine and Physiology had three representatives each. There were 22 male and 7 female participants. There were eight professors among the participants. Five members of the ECs did not answer the first three questions, as they do not review research proposals. The first two paragraphs below describe results only from 24 participants who were members of ERCs.

### Requirement of an information leaflet and a consent form

According to 23 (96%) participants, it is a requirement of the ERC they serve, to submit a copy of the information leaflet and consent form, along with the research proposal. However only seven (29%) reported that their ERC had a standard format for the information leaflet and consent form.

### The quality, of the information leaflets and consent forms that they receive

Opinions on the quality of information leaflets were mixed: 12 (41.3%) felt that they were of a good quality, two (6.8%) that they were of a poor quality and 10 (34.4%) felt that they were a mixture of both good and bad. The interpretation of 'good quality' meant that the basic requirements of information leaflets and consent forms were met, and they were written in simple language that could be understood by lay people. ERC members who felt the quality was bad or a mixture of good and bad gave following reasons; research is not clearly explained, not worded in lay language, the explanations were too technical or too long, providing insufficient information regarding what the subject has to do, the voluntary nature of participation is not clearly stated, inadequate information on potential risks, no assurance on confidentiality, no statement on freedom to withdraw from the research.

### Requirements and components of information leaflet and consent forms

Participant's responses to the question on the requirement of an information leaflet are presented in Table 1.

**Table 1:** Requirements for an information leaflet

**Main themes mentioned by respondents**	**Frequency**	**Percent**
Description of the research project -What one is doing	19	65.5%
Potential risks involved in participating	14	48.3%
Possible benefits of the research project to participant or community	14	48.3%
Methodology -How you're doing it	13	44.8%
Right to withdraw from the research at any time without giving reasons	13	44.8%
Objectives -Why it is done	10	34.5%
Statement assuring voluntary nature of participation	10	34.5%
Assurance of confidentiality	9	31.0%
Stating that refusal to participate will not affect the clinical care	7	24.1%
If any illness that may require intervention is identified, what would be the follow-up action	5	17.2%
Title of the research project	4	13.8%
Who the researchers are	4	13.8%
Contact details of the researchers	4	13.8%
What is expected from research participants	3	10.3%
The time expected to be spent participating in the research	2	6.9%
The source of funding	2	6.9%
Introduction about the research	1	3.4%
Statement saying that it is a research project	1	3.4%

Responses given by the participants for the open-ended question; 'what should be the components of a consent form?' is presented on Table 2.

**Table 2:** Components required for a consent form as generated by the participants

**Main themes mentioned by respondents**	**Frequency**	**Percent**
Statement declaring that the participant clearly understood what the research project is about	15	51.7%
Statement about understanding the right to withdraw from the research at any point	12	41.4%
Statement that the refusal to participate will not affect the right for clinical care	8	27.6%
Space for the name, address and the signature of the participant	8	27.6%
Statement of being aware of the potential risks of participating in the study	8	27.6%
Declaration that the information leaflet has been explained or read thoroughly	6	20.7%
Statement that an assurance of confidentiality was given	6	20.7%
Declaring that the decision to participate is voluntary	5	17.2%
It should be in the appropriate language or all 3 languages used in Sri Lanka	4	13.8%
Statement that they understood the possible benefits of the research	4	13.8%
Statement agreeing to participate or giving consent	4	13.8%
Statement declaring that the participant understood this as a research project	3	10.3%
Statement that the participant understood what is expected from him/her	3	10.3%
Statement either receiving or not receiving any payment for participation	2	6.9%
Name(s) and signature(s) of the researchers involved	3	10.3%
Space for the signature of witnesses	3	10.3%
Title of the research project	1	3.4%
Space to put the date that consent was given	1	3.4%
No need for a separate consent form, it could be a continuation of the information leaflet	1	3.4%

Response to the question 'whether they think that there should be a uniform format for information leaflet and consent form in all studies or can it have different levels of details in different studies?' was divided. 15 (51%) participants believed that there should be a uniform format and 14 (48.3%) disagreed. Six participants who believed there should be a uniform format were from the ERCs that provides a standard format for information leaflets and consent forms. Only one participant from such ERCs disagreed about uniform format.

### Need for ethical approval

The majority, 20 (69%), believed that all research need ethical approval. According to them, downside of reviewing all studies are limitations in human resources and time, [eight (40%)], and lack of capacity [10 (50%)]. Other constraints included the inability to have regular ERC meetings, inadequate participation by members, difficulty in obtaining external expert reviews, insufficient formal training and minimal expertise on ethical review process. The other nine (31%) participants believed that certain types of research could be exempted from ethical review such as audits, non-human subject research, retrospective data analysis, research on samples carried out for routine investigations, and studies where the participants are at the same academic or professional level as researchers.

### Language of the information leaflet and consent form

Twenty participants (69%) reported that the ERCs they serve require information leaflets and consent forms to be available in all three languages used in Sri Lanka (Sinhala, Tamil and English). However, only five (17%) reported reading them in all three languages, 11 (38%) reading both Sinhala and English versions and five (17%) reading only the English version. Nevertheless 26 (90%) agreed on the availability of information leaflets and consent forms in all three languages.

### Written consent for research

Fifteen (52%) believed that not all research requires written consent from the participants.

According to them type of research that can be exempted from consent were non-invasive research where there is no risk for participants, research where opinions and knowledge is inquired on a given issue, research in which very personal details are not asked, research where participants are interviewed at home, and surveys. Animal research, laboratory based studies and research using self-administered questionnaires also could be conducted without written consent. Three participants mentioned that illiterate debilitated or mentally handicapped participants in research study could be exempted from informed written consent.

Then these 15 participants were asked to propose alternative methods to obtain consent. Suggestions included verbal consent (11), implied consent through anonymous questionnaires (two), and thumbprint (one). Some respondents mentioned proxy consent in which permission is obtained from a responsible person of the community, or an independent lawyer on behalf of the participant.

Those who believed that all research required written consent (14; 48%) gave following reasons; to safeguard the rights of participants, patients and researchers, to ensure that participants have understood the research, and for legal reasons.

The number of themes generated for the consent forms (N = 18) appear to be as numerous as for the information leaflet (N = 19). Ten themes (items 2, 3, 5, 7, 8, 9, 11, 12, 14 and 18 from the Table 1 and 2, 3, 5, 7, 8, 10, 12, 13, 15 and 17 from Table 2 are similar) appear in both information leaflet and consent form.

### Comparison with WHO Checklist on informed consent

In this part of the study, the participants' views were obtained on an adapted version of WHO checklist on informed consent. Twenty-seven (93%) participants completed the questionnaire (see Table 3).

**Table 3:** Participant agreement on the WHO checklist on informed consent

**WHO Recommendations**	**Responses by participants**			
	
	**Extremely important**	**Important**	**Not important**	**Not sure**
Should separate consent forms be developed for different levels of questionnaires or procedures?	6(22.2%)	11(40.7%)	6(22.2%)	4(14.8%)
Should the information leaflets written in lay persons' language?	23(85.2%)	0(0%)	2(7.4%)	2(7.4%)
Should it be made clear that the proposed study is a research?	20(74.1%)	7(25.9%)	0(0%)	0(0%)
Should it describe the purpose & duration of the research?	11(40.7%)	15(55.6%)	1(3.7%)	0(0%)
Should it describe the procedures to be carried out?	18(66.7%)	9(33.3%)	0(0%)	0(0%)
Should it provide information on risks & discomforts to participants?	25(92.6%)	2(7.4%)	0(0%)	0(0%)
Should it describe the benefits for the research participants, if any & for others?	14(51.9%)	11(40.7%)	1(3.7%)	1(3.7%)
Should it include the procedures to be followed to ensure confidentiality of the research participants and the information provided by them?	19(70.4%)	7(25.9%)	1(3.7%)	0(0%)
Should it describe the nature of any compensation or reimbursement to be provided?	13(48.1%)	10(37.0%)	1(3.7%)	2(7.4%)
Should it specifically mention that participation is voluntary & refusal to participate will involve no penalty or loss of medical benefits to which the participant was otherwise entitled?	23(85.2%)	3(11.1%)	1(3.7%)	0(0%)
Should it describe alternatives to the participation?	6(22.2%)	8(29.6%)	4(14.8%)	9(33.3%)
Should it provide the name & contact information of a person who can provide more information about the research project at any time?	10(37.0%)	16(59.3%)	0(0%)	1(3.7%)
Should it conclude with a personal statement	14(51.9%)	10(37.0%)	0(0%)	2(7.4%)
Should provision be made for the subjects incapable of reading & signing the written consent form?	16(59.3%)	11(40.7%)	0(0%)	0(0%)
Should provision be made for subjects incapable of giving personal consent?	19(70.4%)	6(22.2%)	0(0%)	1(3.7%)

## Discussion

Majority of the participants acknowledged that the submission of information leaflet and consent form with the study protocol as a requirement in the ERCs they serve. A smaller majority of the participants were of the opinion that all research needs ethical approval. However, they believed lack of adequate capacity and critical mass hampers efficient functioning of ERCs. Increased and continuous training, adequate educational and reference material, increased awareness and attracting more academics will increase the critical mass and increase capacity in existing ERC members.

Because bioethics and ethics review are still at developing stages in Sri Lanka [15], examining and reflecting on informed consent process as done in this study, will contribute to capacity enhancement among ERC members themselves and the review process.

The number of themes generated for the consent form (N = 18) is as many as for the information leaflet (N = 19) and had several overlaps. This may suggest that the consent form should be itemized to reflect the contents covered in the information leaflet. This approach may have an advantage, as the consent form will provide more clarity to what the participant consents for, by including more subcomponents. However, the disadvantage may be that the consent form also becomes similar to the information leaflet.

As Sri Lanka is a multi-religious, multicultural and a multilingual country, any research carried out within the country has to meet certain criteria to satisfy diverse requirements, ethically and otherwise. In this study, participants stressed the need for information leaflets and consent forms to be presented in all three official languages. This is an important recognition of the necessity in a multilingual society. However, it could be an additional burden on already stretched resources available to conduct and review research.

The majority of participants stated that not all research needs written consent. Elaborating on the research types that do not warrant written informed consent most participants mentioned research that has little or no risk to the participants. All the above factors appear to be reflecting on the inadequate resources to review all research.

Additional concern voiced was that written informed consent might limit participation even in research that has minimal-risk [16].

Three ERC members were of the opinion that illiterate, debilitated or mentally handicapped participants need not provide written consent. These groups are highly vulnerable participants in research and needs special protection. Because the questions were pre-agreed and finalized before the interview this controversial statement was not probed further. However, they also proposed alternative methods for obtaining consent. Illiterate participants cannot sign or thumbprint a form they cannot read [17]. Participants' significant other, a clinic nurse who is not involved in the research or a patient representative can be involved during the consent process. Ombudsman structure was proposed by us not only when the participants are vulnerable (debilitated, mentally handicapped or illiterate) but in developing world where there is an exaggerated asymmetry in knowledge and authority exists between researchers and participants [17]. Verbal consent is an alternative but proof of this is an issue. With more than 90% literacy, illiterate research participants in Sri Lanka may be a rarity but due to adverse socioeconomic conditions and free health care, the asymmetry between physician researcher and patient participant will always be an issue

A majority of ERC members rated most components of the WHO checklist as extremely important or important. A significant overlap of themes was noted between the responses to the open-ended questions and the WHO checklist.

Under representation of lay members from ERCs is a limitation in this study. There is a selection bias in the sample with opinion from 29 ERC members who agreed to participate with non-participation by members from three ERCs. Questions on the quality of the information leaflets and consent forms could have been more refined. Using a structured questionnaire without the flexibility of probing the answers in depth was also a drawback in this study. Analysing the qualitative data without using qualitative software (e.g. NVivo) was also a limitation.

In conclusion, our study participants have demonstrated that local standards expressed by them are in par with WHO requirements. The themes generated for the consent form and information leaflet are numerous and similar. This suggests that the consent form needs to reflect the contents covered in the information leaflet.

## Post Script Abbreviations

**ERC**: Ethics Review Committee; **SLAAS**: Sri Lanka Association for the Advancement of Science, **SLTR**: Sri Lankan Twin Registry; **IoP**: Institute of Psychiatry; **WHO**: World Health Organisation.

## Competing interests

The authors declare that they have no competing interests.

## Authors' contributions

AS proposed the initial conceptual framework for the research and was responsible for overall conduct of the project. AS, SS, SH, MP and JM were involved in the protocol design, writing and editing the manuscript. SS, SH, ML, MA, CS contributed to the modification of the protocol, collection and analysis of data including the statistical analysis. AS prepared, the first draft and SH, CS and SS wrote the second draft. All authors were involved in subsequent editing and agreed on the final version.

## Pre-publication history

The pre-publication history for this paper can be accessed here:



## Supplementary Material

Additional file 1 Interview guide. Click here for file
