# Supplementary material for: Informed consent in Sri Lanka: A survey among ethics committee members
Source: BMC Med Ethics. 2008 May 20;9:10. doi: 10.1186/1472-6939-9-10 (PMC2413248; doi:10.1186/1472-6939-9-10)
Supplement: Additional file 1 — Interview guide. [file 1472-6939-9-10-S1.pdf]

Appendix 1. The Interview Guide

Thank the participant first for agreeing to take part in the study and spare his/her valuable time.

Q1. Is it a requirement of your committee to provide a copy of the information leaflet and a consent form, along with the research proposal?

Yes/No

→ Have you got a standard format? Yes/No

→ Have you got a standard format?

If yes obtain a copy

Q2. What do you think about the quality of the information leaflets and consent forms that you receive? **(Ask for his/her general opinion)**

Q3. Tell us more about why you think they are good or bad.

Q4. In your opinion, what should be the components of an information leaflet?

Q5. In your opinion, what should be the components of a consent form?

Q6. a) Do you think that there should be a uniform format for information leaflet and consent form in all studies or can it have different levels of details for different studies?

Yes it should be uniform-

No it shouldn't be uniform-

(e.g.; genetic vs. non-genetic)

b) Can you think of prioritizing its importance depending on the type of the study?

Q7. Does all research need Ethical approval?

Yes / No

What are the constrains of having to review all studies?

If not, what are the types of research that could be exempted?

Q8. Do your ethics review committee Sinhala, Tamil and English versions of the information leaflets and consent forms? Yes/No

Q9. If you have the translations, which one do you read?

If NO go to Q10

Q10. In your opinion, do you think that you need to have Sinhala, Tamil and English versions of the information leaflets and consent forms?

Yes/No

Q11.In your opinion would all research requires written consent from the participant?

Yes/No

Give reasons

a) If no, what are the exceptions? (Probe once they have finished)

b) In such occasions do you propose any other method to get consent?

Q12. Views on WHO recommendations for information leaflets and consent forms.

Appendix 2. Adapted WHO checklist on informed consent

Responses graded as essential or non-essential (in a Scale)]

- Should separate informed consent forms be developed for different levels of questionnaires or procedures?
- Should the information sheet be written in laypersons' language?
- Should it make it clear that the proposed study is research?
- Should it describe the purpose and duration of research?
- Should it describe the procedures to be carried out?
- Should it provide information on the risks and discomforts of participants?
- Should it describe the benefits for the research participants, if any, and for others?
- Should it include the procedures to be followed to ensure confidentiality of the research participants and the information provided by them?
- Should it describe the nature of any compensation or reimbursement to be provided?
- Should it specifically mention that participation is voluntary and refusal to participate (or discontinue participation) will involve no penalty or loss of medical benefits to which the participant was otherwise entitled?
- Should it describe the alternatives to participation?
- Should it provide the name and contact information of a person who can provide more information about the research project at any time?
- Should it conclude with a statement such as: "*I have read the forgoing information, or it has been read to me. I have had the opportunity to ask questions about it and any questions I have been*

asked have been answered to my satisfaction .I consent voluntarily to participate as a subject in this study and understand that I have the right to withdraw from the study at any time without in any way affecting my further medical care"?

- Should provision be made for the subjects incapable of reading and signing the written consent form (e.g. illiterate patient)?
- Should provision be made for subjects incapable of giving personnel consent (e.g. for cultural reasons, children or adolescents less than the legal age for consent in the country, subjects with mental illness, etc)?
